# Study on the Possibilities of Developing Cementitious or Geopolymer Composite Materials with Specific Performances by Exploiting the Photocatalytic Properties of TiO_2_ Nanoparticles

**DOI:** 10.3390/ma16103741

**Published:** 2023-05-15

**Authors:** Andreea Hegyi, Adrian-Victor Lăzărescu, Adrian Alexandru Ciobanu, Brăduţ Alexandru Ionescu, Elvira Grebenişan, Mihail Chira, Carmen Florean, Horaţiu Vermeşan, Vlad Stoian

**Affiliations:** 1 NIRD URBAN-INCERC Cluj-Napoca Branch, 117 Calea Floresti, 400524 Cluj-Napoca, Romania; andreea.hegyi@incerc-cluj.ro (A.H.); bradut.ionescu@incerc-cluj.ro (B.A.I.); elvira.grebenisan@incerc-cluj.ro (E.G.); mihail.chira@incerc-cluj.ro (M.C.); carmen.florean@incerc-cluj.ro (C.F.); 2 NIRD URBAN-INCERC Iaşi Branch, 6 Anton Şesan Street, 700048 Iaşi, Romania; 3 Faculty of Materials and Environmental Engineering, Technical University of Cluj-Napoca, 103–105 Muncii Boulevard, 400641 Cluj-Napoca, Romania; horatiu.vermesan@imadd.utcluj.ro; 4 Department of Microbiology, Facutly of Agriculture, University of Agricultural Sciences and Veterinary Medicine Cluj-Napoca, 3–5 Calea Mănăştur, 400372 Cluj-Napoca, Romania; vlad.stoian@usamvcluj.ro

**Keywords:** self-cleaning cementitious composites, geopolymer, TiO_2_ nanoparticles, physical-mechanical performance, microorganism resistance

## Abstract

Starting from the context of the principles of Sustainable Development and Circular Economy concepts, the paper presents a synthesis of research in the field of the development of materials of interest, such as cementitious composites or alkali-activated geopolymers. Based on the reviewed literature, the influence of compositional or technological factors on the physical-mechanical performance, self-healing capacity and biocidal capacity obtained was analyzed. The inclusion of TiO_2_ nanoparticles in the matrix increase the performances of cementitious composites, producing a self-cleaning capacity and an anti-microbial biocidal mechanism. As an alternative, the self-cleaning capacity can be achieved through geopolymerization, which provides a similar biocidal mechanism. The results of the research carried out indicate the real and growing interest for the development of these materials but also the existence of some elements still controversial or insufficiently analyzed, therefore concluding the need for further research in these areas. The scientific contribution of this study consists of bringing together two apparently distinct research directions in order to identify convergent points, to create a favorable framework for the development of an area of research little addressed so far, namely, the development of innovative building materials by combining improved performance with the possibility of reducing environmental impact, awareness and implementation of the concept of a Circular Economy.

## 1. Introduction

Worldwide, in line with the principles of Sustainable Development and Circular Economy, there is a strong orientation towards reducing the consumption of non-renewable raw materials, increasing sustainability, reducing soil, water and air pollution and, consequently, reducing the volume of waste or identifying possibilities for its recovery. In the construction sector, there is a huge consumption of cement, which is the main raw material in many technological processes specific to this sector [1]. Each ton of cement produced requires between 60 and 130 kg of liquid fuel or its equivalent, depending on the type of cement and the manufacturing process used, and about 110 kWh of electricity [2]. One ton of manufactured cement also releases between 0.8 and 1.1 tons of CO_2_ into the atmosphere as a consequence of fuel combustion and limestone calcination [3]. In 2008 alone, world cement production was about 2.9 billion tons and in 2014, about 4.2 billion tons [4,5]. Producing such a large volume of cement/concrete is directly associated with environmental problems—cement production is responsible for about 5–8% of total carbon dioxide emissions. A “green” alternative to cement-intensive concrete can be the so-called geopolymer concrete [6,7,8].

New binder materials, known as “geopolymers” were introduced in 1987 by Davidovits to describe a group of mineral binders, with a chemical composition similar to natural zeolitic materials, but with an amorphous microstructure. He stated that “binders could be produced by a polymeric reaction of alkali liquids with silicate and aluminium, with source materials of geological origin or with waste materials such as thermal power plant ash” [9]. Geopolymer cements are under development, with research being driven mainly by the need to reduce global CO_2_ emissions. With excellent mechanical properties and strengths in aggressive environments, these materials represent an opportunity for both the environment and engineering, an alternative to traditional technology [10,11]. The concept of Alkali Activated Geopolymer Materials (AAGM) as an alternative to Portland cement has been known since the 1980s. The durability of materials produced by this process has also been demonstrated over the years in Belgium, Finland, Russia, China and recently Australia and the UK. Since the 1990s, most research in the field of alkali-activated geopolymeric materials has focused on the microstructure of these materials and much less on studies on predictions of the engineering properties of these materials, their bearing capacities under different types of stresses, durability, etc., and their actual serviceability. On the other hand, the possibility of producing cementitious composite materials with self-cleaning properties is currently reported worldwide, due to the photocatalytic properties of TiO_2_ nanoparticles used as an addition or as a substitute for certain amount of cement. The first tests were carried out using TiO_2_-enriched white cement-based mixtures. In 1996 the first relevant results were reported, and in 2003, the first large-scale construction of this kind, the Dives in Misericordia church in Rome, Italy, was put into service.

Overall, an analysis of the trend of evolution of research in the field indicates that, if at the beginning, this topic was approached more from the point of view of the effects, respective of the influence of NT on the physical-mechanical performances, at the macro-scopic level, nowadays, the emphasis is put on deepening the study of the phenomenon at the micro-structural level.

Although there are numerous references in the literature, various research groups have approached the problem from different points of view (physico-mechanical aspect, chemical aspect, microstructural aspect, durability or micro-organism resistance aspects, etc.), due to its complexity. It is therefore not possible to identify a pattern on which to build a research programme, given the lack of flexibility and the possibility of recalibration along the way, depending on the intermediate results obtained. This is a direct consequence of the specific character of the locally/regionally available raw materials.

Therefore, the motivation of the research approach aims to contribute to the implementation of the Circular Economy concept by analyzing the possibilities of exploiting waste resources and industrial by-products potentially supplying alumino-silicates, while exploiting the specific performance of TiO_2_ nanoparticles (NT), with the direct destination in the construction industry.

The aim of this work is to carry out a study on the specific performances of NT-containing cementitious composite materials, respectively, alkali-activated geopolymer (GP) materials, as a review of the available literature, thus contributing to the creation of the context favorable to the development of future experimental research.

The scientific contribution of this study consists of bringing together two apparently distinct research directions in order to identify convergent points, to create a favorable framework for the development of an area of research little addressed so far, namely, the development of innovative building materials by combining improved performance with the possibility of reducing environmental impact, awareness and implementation of the concept of the Circular Economy.

The paper is a synthesis analysis of some of the papers published in the last 25 years and is structured on the principle of “mirroring” the positioning of two innovative building materials: cementitious composites with self-cleaning capacity vs. geopolymer composites.

On the one hand, based on references that indicate a better possibility of documentation due to the earlier approach of the field, Section 2 presents a synthesis of research in the field on the possibility of inducing self-healing capacity and increasing resistance to the action of biological agents in cementitious composites by exploiting the specific properties of NT. Other influences of NT use on cementitious composites are also presented, i.e., effects on the physical-mechanical performance of both fresh and hardened cementitious composites. Finally, for a good argumentation of the specific innovative element characteristic of these cementitious composites, aspects related to the NT specific photoactivation phenomenon and the specific mechanisms leading to the self-healing and biocidal performance are presented.

On the other hand, in Section 3, aspects related to geopolymer materials are addressed, touching both specific elements of the geopolymerization reaction and specific elements of the physical-mechanical performances. The field of self-healing geopolymers is still poorly represented at the level of reports in the literature, there is still a strong interest in understanding the mechanism and kinetics of the geopolymerization phenomenon in general, the influencing factors and, only subsequently, the impact that the introduction of nano-TiO_2_ has on the performance of this type of material.

Therefore, the challenge of carrying out such a synthesis study included identifying as many bibliographical sources as possible for the two directions addressed. If in the case of cementitious composites, the mechanism of hydration-hydrolysis reactions of cement can be said to be, at present, amply documented. In the case of geopolymers, there are still many aspects that need clarification. Moreover, in the case of the influence of NT on the physical-mechanical performance, durability, self-healing and biocidal capacity, for cementitious composites there is a wealth of reported studies. There is already a history, in contrast to the situation of geopolymer materials which can be said to be still in their infancy from this point of view. However, it should be specified that the purpose of this work and the way of documentation was aimed to analyze strictly on the field of cementitious composites produced with Portland cement vs. geopolymer binders, with NT content, without analyzing other material, e.g., non-Portland cements, lime mortars, lime and cement mortars and hydraulic lime mortars modified with TiO_2_.

## 2. Cementitious Composites with Self-Cleaning Capacity

Numerous studies indicate that the introduction of nanoparticles, including TiO_2_ nanoparticles (NT) in the mixture of a cementitious matrix, has beneficial effects on the physico-mechanical properties and durability and induces some specific performances resulting from photoactivation [12,13,14,15,16,17,18]. From the outset, the literature indicates two ways of exploiting the self-cleaning and biocidal effects of NT-specific performance: by applying an NT-containing film to the surface, or by developing NT-containing composite material in mass. In this work the study focused on the documentation and analysis of these NT-containing composite materials in the matrix.

In general, for use, NT is found as a mixture of rutile and anatase (both being crystallographic forms of TiO_2_). There is also the situation where the crystallographic form anatase is predominant (over 90%), and the literature recommends that TiO_2_ nanoparticles be added dry, by direct mixing with cement powder, followed by the addition of hydration water. Water does not chemically react with any crystallographic form of titanium dioxide, nor does a chemical reaction occur between the photosensitive nanoparticles and the hydration-hydrolysis phases of the cement, therefore the hydration-hydrolysis reactions are not chemically influenced [19], but, as will be seen in the following, their kinetics will be influenced.

However, the size and particle size distribution of the TiO_2_ powder used influences the cementitious composite material, so research has been carried out at the University of Milan, Italy, using TiO_2_ powder with micrometric size, m-TiO_2_, and nanometric size, n-TiO_2_, respectively. The results showed that there are advantages and disadvantages in both cases [19]. Thus, the dispersion and distribution in the matrix is more convenient when using micrometer-sized granules, while nanometer-sized ones tend to agglomerate, thus reducing the overall reactive surface available for the initiation of photocatalytic reactions. On the other hand, the use of nanometric granules is advantageous and preferable, although more difficult, because, under conditions of homogeneous, satisfactory dispersion, the composite will have a better capacity for adsorption of pollutant oxides (NOx, SOx, dust, exhaust fumes), which can easily penetrate the agglomerations of nanoparticles, and the hydrophilicity effect is more intense, but the research carried out so far has not shown a definite influence of particle size on the efficiency of TiO_2_-enriched cement matrix in terms of the decomposition capacity of pollutant molecules and dirt particles [20]. Research to date has explored both micrometer- and nanometer-sized TiO_2_. However, although the photoactivation phenomenon of nano-TiO_2_ is more obvious in terms of the properties induced in the cementitious matrix in which it is embedded, there is also the possibility of using micro-TiO_2_, each of which has advantages and disadvantages. On the one hand, nanoparticles raise the problem of homogeneous dispersion in the mass, which is easier to achieve if the grain size is within micro limits. On the other hand, the physico-mechanical characteristics, the hydrophilicity of the surface, the decomposition capacity of organic/dirt particles and the biocidal effect are lower when using micro-sized TiO_2_ granules. A possible solution to improve the dispersion rate of TiO_2_ particles would be to use dispersing agents and superplasticizers.

However, the influence of the qualitative and quantitative parameters of the photoactivation light should not be neglected either, as research has shown that there are differences, even when keeping all the designed preparation parameters, whether the composite preparation takes place in artificial light, natural light or darkness, or in the same type of light but with different intensities [19,21].

The unanimously accepted conclusion is that, for each case of a particular cementitious composite, it is necessary to determine the optimal amount of TiO_2_ nanoparticles used in the composite, as an additional amount is not economically justified and very often may negatively influence some parameters of the cementitious composite matrix enriched with TiO_2_ nanoparticles.

### 2.1. Influence on the Physical-Mechanical Characteristics of Fresh Cementitious Composites

Given the complex structure of cementitious composites and the hydration-hydrolysis mechanisms underlying the formation of this structure, i.e., the C-S-H gel formation phase, the introduction of NT involves a number of difficulties, especially in terms of their tendency to agglomerate, which reduces the potential to obtain a homogeneous dispersion in the cementitious mass. Therefore, this aspect induces some modifications in the C-S-H gel and, implicitly, on the physico-mechanical properties of the whole composite [22]. According to reported research, one of the biggest challenges is the uniform and homogeneous dispersion of NT in the composite matrix [22,23,24]. This problem has been analyzed by applying various dispersion methods, i.e., the success of water preparation using plasticizers and superplasticizers [25,26] or the use of ultrasonic waves [27]. Thus, Perez-Nicolas et al. [28] have shown the possibility of obtaining a homogeneous dispersion using polycarboxylate-based superplasticizers that effectively prevented NT agglomeration. Similarly, Zhao et al. [29] report results of research on engineered cementitious composites (ECC) with NT, but also with polyvinyl alcohol fibers (PVA), in the preparation of which polycarboxylate-based superplasticizers were also used, an additive which again proved effective. As reported by Perez-Nicolas et al. [28], plasticizers and superplasticizers are frequently used in the field of cementitious composites due to their mechanism of reducing water requirements and facilitating homogeneity through a mechanism based on the induction of these molecules which exhibit a dispersing action between cement particles due their electrostatic repulsion and/or steric hindrance effects [30,31]. Their research [28], however, focused on coatings made with water dispersions of different nanoparticles of photocatalytic additives (titania and titania doped with iron and vanadium), analyzing the efficacy of three types of polycarboxylate-based polymers, synthesized in the authors’ research, demonstrated the effect of preventing agglomeration of nanoparticles, improving aqueous dispersions, unlike a fourth, commercial superplasticizer, which led to the formation of NT agglomerates. Therefore, not only the use of superplasticizers is important for the production of homogeneously dispersed NT cementitious composites and therefore with uniform and homogeneous properties, but also the type of superplasticizer, especially as this technique disperses nanoparticles in the preparation water, as a raw material preparation phase prior to the preparation of the cementitious composite, is also frequently encountered when considering the production of these composites with nanoparticles dispersed in the mass, the homogeneous dispersion problem also being encountered in the case of SiO_2_ nanoparticles [32]. Other research has addressed this issue in a different way, e.g., the possibility of coating the NT granule with nano-SiO_2_ [26] was investigated, leading to a reactive powder which, following microstructural analysis, was found to be uniformly dispersed in the composite matrix.

Regarding the fresh self-cleaning cementitious composite, following the embedding of TiO_2_ nanoparticles (NT), the following were observed: increased water requirement to reach standard consistency, decreased workability, decreased initial and final setting time, accelerated hydration-hydrolysis processes, increased heat of hydration and cement hydration rate, reduced porosity of the cement paste due to changes in pore size and distribution, altered size and orientation of cement hydration product crystals and formation of a larger amount of C-H-S calcium hydrosilicate gel [33,34,35]. Thus, Nazari et al. showed that partial replacement of cement, or addition of nano-TiO_2_, contributes to the reduction in setting time and intensification of cement hydration-hydrolysis processes, findings that correlate with those reported by Lee et al. and Pimenta Teixeira [12,15,16,17,36,37,38,39]. The phenomena can be explained both because TiO_2_ nanoparticles function as possible concentrating nuclei for the hydration products and on the catalytic effect they have on the cement hydration reaction. The first effect observed, as early as the preparation phase of cement-based mortars and concrete, is an increase in the water required to reach the standard consistency [33].

In terms of workability, it can be said that it decreases with an increasing percentage of TiO_2_ nanoparticles, introduced into the cement paste either as an addition or as a substitution of a part of OPC [40,41,42,43,44,45,46,47], a similar issue being reported regarding the flowability of the composite mixture [48]. These phenomena are reported in the literature for a percentage amount of NT used, varying in the range of 0.5–10% relative to the amount of OPC. At the same time, the literature indicates that the workability of nano-TiO_2_-containing cementitious composites does not vary in proportion to the amount of nanoparticles used and, moreover, different reductions in workability are identified even for the same amount of nano-TiO_2_ [48]. This phenomenon can be attributed to differences in terms of the type of cement used, its oxide composition, water/cement ratio, the type of additive used and in terms of preparation conditions.

Thus, Salemi et al. [41] indicate a reduction of up to 50% in the flowability of the composite when using a maximum of 2% NT; Nazari et al. [42] indicate a reduction in workability of about 25% when replacing 2% OPC with NT for a water/cement ratio of 0.4 and Li et al. [43,44] report workability reductions of over 50% when replacing a maximum of 3% OPC with NT and even over 70% when using 5% NT. In contrast, Meng et al. [47] report a workability reduction of only 21% and 40%, respectively, under the conditions of substitution of OPC with 5% and 10% NT, respectively, for a water/cement ratio of 0.5. Other research [34] indicated that substitution of OPC with max. 1% NT, mass ratio, does not significantly influence the flowability of cement-based mortar. Therefore, the existence of this controversy, the impossibility of a quantifiable prediction of how the workability of the fresh composite will vary depending only on the amount of NT used, induces the need for further experimental research and preliminary case-by-case customized analyses specific to the raw materials used before designing any nano-TiO_2_-containing cementitious composite mixtures.

In terms of setting time, research has shown that from 0.5% to 10% NT content, the initial and final setting times decrease with increasing NT content, but at the same time, the importance of NT type on these parameters is also indicated, i.e., rutile and anatase concentration, both of which are crystallization forms of TiO_2_ [49,50,51]. Further research has shown increased heat of hydration and increased hydration rate of OPC as well as change in the structural orientation of CH crystals, i.e., their size [52]. Lee and Kurtis [53] showed that the intensification of hydration processes, manifested by increased heat, hydration rate and degree of hydration of OPC granules occurs during more than 3 days after preparation of the cementitious composite. In agreement with them, Chen et al. [37] indicated the intensification of the hydration process of OPC granule in the present NT, with an increase in the hydration heat especially in the first 30 h after preparation, i.e., 22 h after preparation as indicated by Senff et al. [54].

Additionally, Nazari and Riahi [55], Jalal et al. [56], Jayapalan et al. [57,58], Kurikara and Maruyama [59], Baoguo et al. [60], Sakthivel et al. [61], Khataee et al. [62] and Hamidi et al. [24] confirm the acceleration of OPC hydration processes in the presence of NT, for amounts of up to 5% NT as a substitute of OPC used, the general result being a gain in terms of mechanical strength at early ages, a phenomenon also influenced by the rutile–anatase composition of NT, respectively, the grain size distribution of NT.

The decrease in workability and grip type, concomitant with the increase in hydration heat and hydration rate of OPC leads to a possible explanation: due to the significant increase in the specific surface area of the particles in the mixture used for the preparation of the composite, a large, specific surface area of NT induces higher and faster water consumption but also an increased reactivity overall, NT functioning as a catalytic effect of the hydration-hydrolysis processes and functioning as potential accumulation nuclei of hydration products.

### 2.2. Influence on the Physical-Mechanical Characteristics of Cementitious Composites in the Hardened State

Nazari et al. showed that partial replacement of OPC or addition into the composite cement matrix of NT contributes to densification of the composite material, reduction and resizing of porosity and increase in mechanical strengths, findings that correlate with those reported by Lee et al., Pimenta Teixeira and Feng et al. [12,15,16,17,36,37,38,39]. Up to a certain dosage of TiO_2_ nanoparticles, the dosage limit above which a reduction in these parameters occurs, also in agreement with those reported by Zhang and Li. [43,44].

Thus, as presented by Rashad [55], referring to Nazari et al. [15,16,17,53], shows that partial replacement of OPC with 0.5–2% NT, for a water/cement ratio of 0.4, induces an increase in mechanical strength at 28 days of specimens, by 15.91–25.00% for flexural strength, by 5.55–66.67% for splitting tensile strength and by 6.79–17.93% for compressive strength. However, it should be noted that these variations are not linearly increasing. The variation curves showing a maximum for the case of OPC substitution with 1% NT, after which the slope of increase in mechanical performance follows a downward trend. This behavior could be explained precisely based on the phenomena that are proven to occur in the preparation and early phase of the cementitious composite, namely, the consumption of a quantity of the mixing water by the NT and the acceleration of the hydration-hydrolysis processes of the cement granule in the first approximately 72 h after casting. It could thus be said that substitution of OPC with an amount of more than 1% NT could have better results if additional water was added to the mix.

In agreement with Nazari et al., Li et al. [24] report an increase in flexural strength of 10.27% and 2.93%, respectively, for cementitious composites at 28 days of age for replacement of OPC with 1% and 3% NT under the condition of maintaining the water/cement ratio of 0.42. In terms of compressive strength, Li et al. [24] but also Zhang and Li [63], under the same conditions of water/cement ratio of 0.42 and testing at the age of 28 days, indicate an increase of the parameter by 18.03% for 1% NT, 12.76% for 3% NT and 1.55% for 5% NT. Agreement with the optimal NT percentage indicated by Nazari et al. is observed, i.e., 1% mass percentage, an amount of NT that substitutes OPC without inducing an increase in the preparation water requirement and that provides maximum benefit.

**Figure 1:**
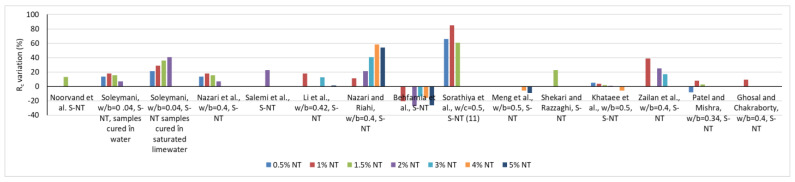
Variation of the compressive strength based on several research [15,16,17,18,41,43,44,47,48,62,64,66,67,68,69,70,71].

**Figure 2:**
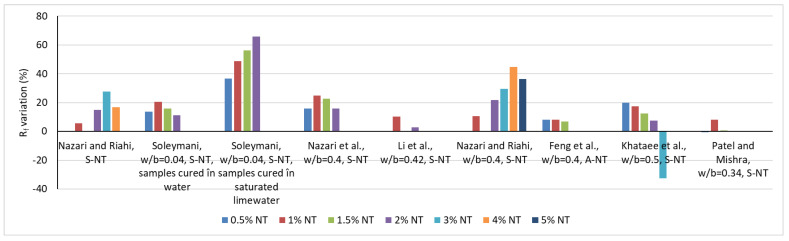
Variation of the flexural strength based on several research [15,16,17,24,25,43,44,62,66,67,70].

In contrast to general trends, Behfarnia et al. [64], Sandu et al. [65] and Meng et al. [47] report reductions in compressive strength with the introduction of NT into the cementitious composite. A series of reported results on the variation of compressive strength as a function of the amount of NT substituting OPC is presented in Figure 1. Similarly, the variation of the tensile strength is shown in Figure 2.

**Figure 3:**
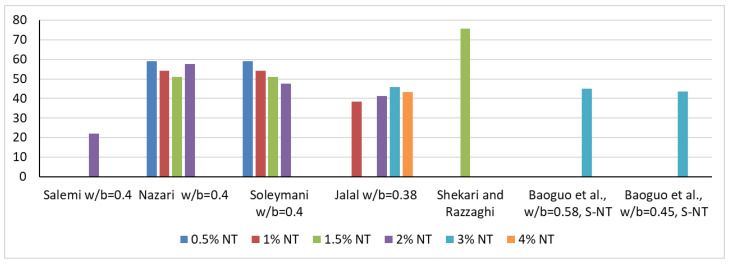
Water absorption of the samples according to several research [15,16,17,40,41,69].

In terms of internal structure, NT causes a reduction in open porosity, which will also lead to a reduction in water absorption. Thus, Salemi et al. [41], in agreement with Nazari [72], indicate that the introduction of 2% NT, respectively, 0.5%, 1%, 1.5% and 2%, into the cementitious composite for specimens at 28 days of age, causes a reduction in water absorption by 22%, 59.1%, 54.1%, 51.1% and 47.5%, as shown in Figure 3. Regarding the resistance to chloride ion penetration, Hi and Shi [73], in agreement with Li et al. [74], indicate a reduction in the penetration rate as a result of OPC substitution, i.e., the addition of 1% NT, a phenomenon that can also be explained by a reduction in the porosity of the composite. If a simple extension of these improvements in physical-mechanical performance is made, it can be appreciated that a foreseeable consequence of the introduction of NT in cementitious composites will also be an improvement in wear resistance, frost-thaw resistance, essential elements of the general durability of these materials, as in fact indicated by Li et al. [44], Hassan et al. [75] or Farzadnia et al. [76].

In terms of influence on physical-mechanical performance, in accordance with the literature, the identification of the optimal dosage of TiO_2_ nanoparticles introduced into the cementitious composite matrix is still a controversial parameter, influenced by several parameters: characteristics of the other raw materials, water/cement ratio, conditioning conditions and test age, temperature, mode of introduction (mixing in the dry or wet phase, introduction as an addition or substitute of OPC) to the preparation, knowing that the photocatalytic activity, expected in these TiO_2_ cementitious materials, is critical for this choice. Too much photocatalyst often leads to electron-hole coupling which reduces the efficiency. Furthermore, Feng et al. [77] identify structural changes present in the cement paste that could explain the evolution of the other physico-mechanical properties, in particular, mechanical strengths, density and porosity. The authors, starting from what is known, namely, that in Portland cement paste (OPC), through hydration reactions of the cement granule, the formation of hydrated calcium silicate (C-S-H) takes place which, in the hardened phase, becomes C_3_S with a Ca/Si ratio in the range 1.25–2.1. Consider, in accordance with the literature, that in the hardened cement matrix this C-S-H divides into two types of formations, one type where the silicate anions are entirely monomeric and the other where a linear silicate chain is present in the tobermorite. With the introduction of 1% (mass percentage) TiO_2_ nanoparticles and ensuring a good dispersion in the matrix, a series of micro and nanostructural changes are achieved: reduction in microcracks and internal defects in the cement paste, obtaining a densification of the material, reduction in nanoroughness concomitant with the formation of nanoprecipitated, needle-shaped hydration products that will function in the reinforced composite matrix as a dispersed nano-reinforcement. Comparative analysis of the influence of NT both in cementitious-based matrices (hydraulic binders) vs. lime-based composites (non-hydraulic binders) showed on the one hand that the photocatalytic activity of TiO_2_ is not influenced by the type of binder, as both materials are porous and NT acts by filling these pores, and on the other hand that this positioning of NT in the composite matrix will lead to improved physico-mechanical performance [22]. TiO_2_ will act as a filler for these pores and make it possible to absorb NT in the case of hydration products and, at the same time, reduce the available surface area of the photocatalyst. Furthermore, the literature shows that an increase in electron-hole recombination can occur for adsorbed species [78,79]. In addition, as the material ages, an increase in volume of almost 10% is observed, due to carbonation. This phenomenon leads to a decrease in capillary adsorption and precipitation of calcium carbonate. These precipitates will act as a barrier and decrease the TiO_2_ capacity [80,81].

### 2.3. Influence of the Introduction of TiO_2_ Nanoparticles into Cementitious Composites on Their Resistance to the Action of Microorganisms—Self-Cleaning Capacity and Biocidal Mechanism

For the first time, in 1985, Matsunaga et al. [82] demonstrated the photocatalytic cell killing mechanism of *Saccharomyces cerevisiae* (yeast), *Lactobacillus acidophilus* and *Escherichia coli* (bacteria) and *Chlorella vulgaris* (green algae) in water treated with TiO_2_ nanoparticles [82].

It is now known that the biocidal mechanism and self-cleaning capacity of composite surfaces containing TiO_2_ nanoparticles is the result of two mechanisms, that of superhydrophilicity and that of degradation, destruction of molecules of an organic nature, therefore, implicitly of the cells of microorganisms these having a structure of an organic nature [18].

**Figure 4:**
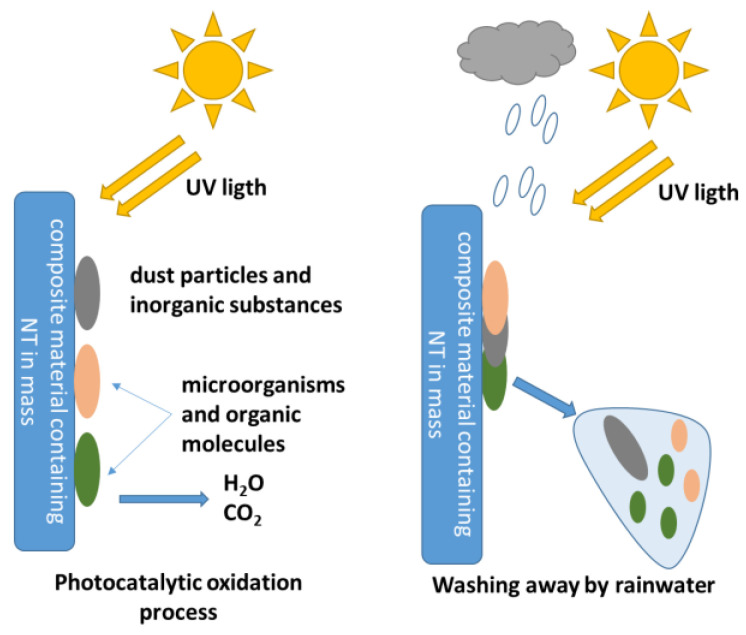
Schematic representation of the self-cleaning phenomenon of composite cementitious surfaces containing TiO_2_ nanoparticles.

If in the case of inorganic substances adhering to the composite surface, the superhydrophilicity mechanism is sufficient for the manifestation of the self-cleaning capacity (the water film picks up and washes the inorganic particles from the composite surface). In the case of organic substances, large-volume organic molecules and microorganism cells, it is necessary that this superhydrophilicity is complemented by the degradation capacity of the organic molecules and/or cells. The schematic representation of the self-cleaning phenomenon of composite cementitious surfaces containing TiO_2_ nanoparticles is presented in Figure 4.

An experimental test method based on a simple principle but demonstrating the accumulation of the two mechanisms is the decolorization method for *Rhodamine B* (RhB), an organic substance, N-9-(2-Carboxyphenyl)-6-(diethylamino)-N,N-diethyl-3H-xanthen-3-iminium chloride, which is characterized by an intense pink-magenta coloring. The principle of the method consists of the fact that, by applying a spot of RhB solution on a cementitious composite surface with TiO_2_ nanoparticles, subsequently, under the influence of UV radiation, with or without the accumulation of washing steps, discoloration is rapidly observed, as a result of the degradation of the organic molecule (oxido-reduction reactions) and, in the case of the existence of the washing step, the uptake of the reaction products by the water film developed on the cement composite surface which has become superhydrophilic [83]. A similar method has been identified in the literature as using *Methylene blue*, Methylthioninium chloride with the formula C_16_H_18_ClN_3_S as the organic staining substance [84], with the mention that the advantage of the RhB staining method is primarily represented by the more intense highlighting of the stain and the bleaching effect.

**Figure 5:**
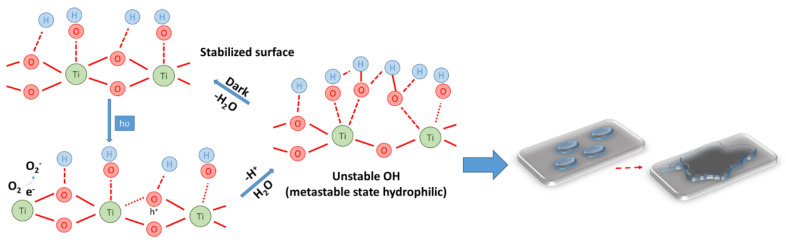
Schematic representation of the superhydrophilicity mechanism.

The mechanism underlying the increase in hydrophilicity of TiO_2_ nanoparticle-containing cementitious composite surfaces under UV exposure can be explained based on the increase in hydroxyl (OH^−^) groups identified by X-ray photoemission spectroscopy (XPS), Fourier transform infrared spectroscopy (FTIR) or nuclear magnetic resonance (NMR) [85,86,87,88,89,90]. The surface transition, under the influence of UV radiation, into a thermodynamically metastable state is the result of the coexistence of two molecular forms of water: molecular water and dissociated water. In general, under the action of UV rays, titanium dioxide being a semiconductor with a band gap of about 3.0 eV, by absorbing energy, generates electrons (e^−^) and voids (h^+^). Electrons tend to reduce Ti(IV) cations to Ti(III) ions, and voids oxidize O^2−^ anions. This process will release oxygen, creating vacancies on the surface of the titanium dioxide, vacancies that allow the water molecules to bind with the release of hydroxyl groups (OH^−^). Additionally, in the case of TiO_2_-containing cementitious composite surfaces, the literature also indicates [85,86,87,91] that photogenerated (h^+^) vacancies (voids) cause the bond length within the TiO_2_ network to increase, bringing the surface into a metastable state allowing adsorption of molecular water, simultaneously with the formation of new hydroxyl groups and the release of a proton (Equations (1)–(3), Figure 5). Research has shown that these generated hydroxyl groups are less thermodynamically stable, therefore, the surface will allow flattening of the water droplet to cover a larger area for stabilization purposes [85,86,87,91,92,93,94], thus achieving the effect of superhydrophilicity, i.e., the water that will arrive on the photoactivated composite surface will no longer form individual droplets, but lamellae that favor the uptake of impurities (Figure 5).

TiO_2_ + hʋ → e^−^ + h^+^ (1)

e^−^ + Ti^4+^ →Ti^3+^ (2)

4h^+^ + 4O^2−^ → 2O_2_ (3)

In conjunction with the development of superhydrophilicity, using the energy provided by UV radiation, which is greater than the valence band gap of TiO_2_, pairs of electrons (e^−^) and voids (h^+^) are generated, which react with O_2_ and H_2_O to form anionic radicals (O^2−^) and (OH). These oxidative species (h^+^,∙(O^2−^) and (OH)) are all highly reactive, contributing to the destruction of microorganism cells [95] according to a sequence of chemical reactions shown in Equations (4)–(9). This mechanism of organic cell destruction is coupled with the superhydrophilicity mechanism specific to the surfaces of TiO_2_ nanoparticle-enriched cementitious composites, so that after the initiation of destruction at the cellular level, the organic residue, not yet completely degraded to CO_2_ and H_2_O, can be more quickly and easily removed from the surface by simple washing (under rainy conditions), leading to increased performance in terms of surface safety and hygiene.

TiO_2_ + hν→TiO_2_ (e^−^*_cb_* + h^+^*_vb_*) (4)

O_2_ + e^−^*_cb_*→O^2−^ (5)

H_2_O + h^+^*_vb_*→•OH + H^+^ (6)

•OH + •OH→H_2_O_2_ (7)

O^2−·^ + H_2_O_2_→•OH + OH^−^ + O_2_ (8)

•OH + Organic + O_2_→CO_2_ + H_2_O (9)

Subsequent research has examined hypotheses regarding the biocidal effect indicating that the cell membrane is photocatalytically destroyed in *Escherichia coli*, as reported by Sunada et al. [96]. The same results have been also supported by reports by Oguma et al. [97], who propose a destruction mechanism explained by both cell wall destruction and induction of cellular disruption following contact of the microorganism with TiO_2_, while Saito et al. [98] propose a hypothesis of a destruction mechanism explained by inhibition of bacterial cell respiration function once in contact with TiO_2_. Research indicates a wavelength of incident TiO_2_ photoactivating radiation in the range 320–400 nm that produces strong destruction at the cellular level [99].

Gogniat et al. [100] showed that the adsorption capacity of TiO_2_ in contact with the cell wall is positively correlated with the biocidal effect and adsorption was consistently associated with a reduction in bacterial membrane integrity. The authors indicate the hypothesis that adsorption of cells onto photocatalyzed TiO_2_ is followed by loss of membrane integrity, which is key to the biocidal effect.

Mazurkova et al. [101], analyzing the effect of photocatalyzed TiO_2_ nanoparticles on the *Influenza* virus, showed that after 15 min of incubation, nanoparticles adhered to the outer surface of the virus, the surface spinules of the virus were glued together, and the outer membrane, lipoprotein in nature, was ruptured. The degree of destruction in the influenza virus increased after 30 min of testing, and after 1–5 h of incubation, the virus was destroyed. The researchers indicate that the biocidal effect is dependent on the existence or not of photoactivation, the type of incident photoactivating radiation (natural light, UV rays) the duration of exposure/incubation, the viral concentration and the concentration of nano-TiO_2_, but in all cases, this effect was recorded sooner or later.

In support of the biocidal effect argument, tests by Adams et al. [102] showed that the concentration of *Bacillus subtilis* and *Escherichia coli* was reduced 3.7 times, respectively, 2 times, upon contact with a nano-TiO_2_ suspension under photoactivation conditions under natural light. A similar effect on *Bacillus subtilis* was also reported by Armelao et al. [103].

Research by Dedkova et al. [104] on samples of kaolinic composite material enriched with TiO_2_ nanoparticles also confirmed the biocidal effect for *Escherichia coli*, *Enterococus faecalis* and *Pseudomonas aeruginosa* after 2 days of exposure to artificial light, reporting also in agreement with Gurr [105], who estimated that the antibacterial effect of TiO_2_ composites manifests itself in the presence of natural light, without necessarily requiring additional photoactivation with incident radiation with wavelength exclusively in the UV spectrum.

As for cementitious composites enriched with 3% and 5% TiO_2_ nanoparticles, it has been shown that the viability rate of *E-coli* bacteria is reduced after 24 h by 60% to 70%, compared to the situation without TiO_2_ nanoparticles, according to the study reported by Hamdany [106]. The results of this research agree with reports by Davidson et al. [107], Lorenzeti et al. [108] and Peng et al. [109]. The explanation for this ability would be in line also with reports by Daly et al. [110], Carre et al. [111] and Kubacka et al. [112], which confirm that by the formation of free radicals and strongly oxidized anions by photoactivation of nano-TiO_2_ (OH● and O^2−^) at the cellular level, the cell membrane is ruptured and plasma components such as DNA, RNA, lipids and proteins are destroyed.

In support of these hypotheses are also the results of pilot research by Huang et al. [113], who showed that metals such as Ag and Cu enriched with TiO_2_ have the ability to reduce the survival rate of *Aspergillius Niger* spores under conditions of lack of light radiation and even more so under UV irradiation, respectively, UV irradiation and presence of ozone. Yadav et al. [114] summarized the reports of research results in the field, pointing out that microorganisms are destroyed when they reach the photocatalytic surface, the effect being demonstrated on both Gram-positive and Gram-negative bacteria, endospores, fungi, algae and viruses.

Similar results supporting the antimicrobial character imparted by TiO_2_ nanoparticles to composite cementitious surfaces have been presented by numerous studies [90,115,116,117,118], showing that cementitious composites with 10 wt% TiO_2_ powder reduce algal growth on the surface by 66% compared to cementitious surfaces without nanoparticle content.

Regarding the ability of different building materials to resist microorganism attack, the most convenient method is the one adapted from the antibiogram method used in medicine and known as the halo inhibition method [119] or the Kirby–Bauer method, currently standardized according to AATCC TM147 and AATCC TM30.

The need for uniform evaluation of the behavior of various materials to the action of micro-organisms has led to the development of dedicated method standards as well as non-standardized but frequently used methods. Thus, method standards for qualitative assessment and method standards for quantitative assessment are known [120]:

- ASTM E2149—presents a quantitative method for evaluating the behavior of irregular surfaces to bacterial action. The principle of the method is to immerse the material in a suspension with a known concentration of bacteria and to follow the evolution of this concentration over time. The antimicrobial activity of the material is considered positive when the concentration of the bacterial suspension is significantly reduced [121];
- ASTM E2180—presents a quantitative method for evaluating the behavior of hydrophobic surfaces to bacterial action. The principle of the method consists of making a pseudo-film of nutrient medium on the surface of the material, on which bacteria are inoculated in a suspension of known concentration and monitoring the evolution of the concentration compared to a control [122];
- ISO 22196—presents a quantitative method. The principle of this method is also to follow the variation of the concentration of the bacterial suspension inoculated on a nutrient medium [123];
- ASTM E1428—presents a qualitative method featuring the so-called “pink spot test”, in which an inoculation with *Streptoverticullium* reticulum is used and the appearance of pink spots on the surface of the tested material is observed [124];
- STAS 12718/1989 offers the possibility of semi-quantitative quantification of the microbiological load of the system, providing a quantification grid as follows: 0(−) no growth (sterile); 1(+) 1–10 colonies of microorganisms; 2(++) more than 10 colonies of microorganisms; 3(+++) areas with confluent colonies; 4(++++) growth over the whole surface area [125];
- ISO 27447—presents a method for evaluating the antibacterial activity of semiconducting photocatalytic materials, can be applied for the analysis of some ceramic, photocatalytic materials but not for permeable or rough materials [126].

Although the potential benefits of TiO_2_ nanoparticles have been known since 1921 [127], the ability to destroy microorganisms was not documented until over 60 years ago, starting with Matsunaga et al., in 1985 [128,129,130]. Currently, it is known that the anti-algal and antifungal activity of nano-TiO_2_ is evident in 11 genera of filamentous fungi, 3 yeasts, 2 amoebae, 1 apicomplexan, 1 diplomonad, 1 ciliate and 7 algae, including 1 diatom, with fungal spores being generally more resistant than vegetative forms [131,132,133,134,135,136,137,138,139,140,141]. Sassolini et al. [142] considered the realization of surfaces from TiO_2_ nanoparticle-enriched cementitious materials as a passive form of safety technology to increase safety in biological, radiological and nuclear (CBRN) accidents.

## 3. Geopolymer Composites with Self-Cleaning Capability

By geopolymer, it is meant that type of amorphous, alumino-silicate cementitious material which can be synthesized by the polycondensation reaction between a geopolymeric material and alkali polysilicates. This process is called geopolymerization [143]. This innovative technology allows for the transformation of alumino-silicate materials into products called geopolymers or inorganic polymers. Geopolymers, therefore, represent a material developed as an environmentally friendly alternative for the construction industry, but also as a solution for exploitation, reintroduction into the economic circuit of some industrial wastes and by-products, the most common being fly ash, slag kaolin and metakaolin mostly activated with alkaline solutions based on Na_2_SiO_3_ in combination with NaOH [8,144,145,146,147].

**Figure 6:**
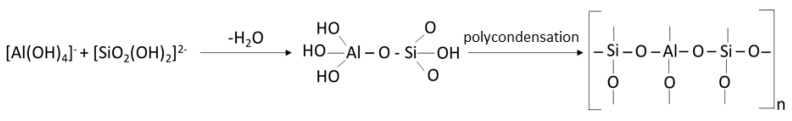
Geopolymer polycondensation process [152].

In 1979, Davidovits described this new family of materials, with amatrix based on a Si-O-Al-O structure, by alternating tetrahedra of SiO_4_ and AlO_4_, joined together in three directions with all oxygen atoms, calling them geopolymer materials [148]. In a simplified method, it can be stated that geopolymers can be synthesized by alkaline activation of materials that are rich in SiO_2_ and Al_2_O_3_ [149]. Although the whole process is still lacking consistent data, there is concrete information that in geopolymerization mechanisms, the dissolution of aluminium (Al) and silicon (Si) in an alkaline medium occurs, followed by the transport (orientation) of the dissolved elements and then the polycondensation phenomenon, as a result of which a 3D network of alumino-silicate structures is formed [150]. In 1999, Palomo proposed that pozzolanic materials (blast furnace slag, thermal power plant ash) can be activated “using alkaline liquids to form a binder and totally replace the use of Portland cement in concrete production” [151]. When the two components of the geopolymer material (reactive solids and alkaline solution) react, an alumino-silicate network is formed, resulting in a hard, water-resistant product, the geopolymerization process can be expressed according to the following sequence of reactions (Equation (10), Figure 6 and Equation (11)) [152]:

2SiO_2_·Al_2_O_3_ + 3OH^−^ + 3H_2_O → 2[Al(OH)_4_]^−^ + [SiO_2_(OH)_2_]^2−^ (10)

The empirical formula for the whole polymerization product is shown in Equation (11) [153]:

Mn{-(SiO_2_)z-AlO_2_}n,wH_2_O (11)

where M—represents the alkali element (which can be: K (potassium); Na (sodium); Ca (calcium)), the symbol “-” indicates the presence of a bond; n—represents the degree of polycondensation (or polymerization); z—is the Si/Al ratio which can be −1, 2, 3 or higher, up to 32.

**Figure 7:**
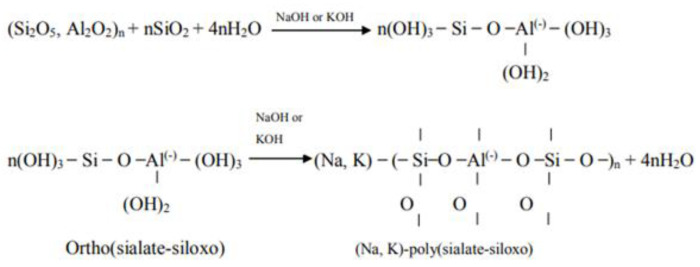
Geopolymerization process according to Wallah [154].

Wallah [154] presented a more detailed equation showing the process by which the powder particles dissolve in alkali to produce the reactant product. In this equation, the reaction of aluminosilicate, hydroxide (Na, K), water and silicate in gel form is shown, followed by the dehydroxylation process to form a network of geopolymers. The reaction is presented in Figure 7.

According to studies, it is known that the reaction of alumino-silicate materials in a strongly alkaline environment first causes the Si-O-Si bonds to break, subsequently, new phases are produced, and the formation mechanism appears to be synthesis via solution. The most important part of this process is the penetration of Al atoms into the original Si-O-Si structure and the formation of alumino-silicate gels. The composition of these gels can be characterized by Figure 7. The C-S-H and C-A-H phases may also originate from their direct dependence on the chemical composition of the materials used and the conditions of reaction production [155]. Additionally, the concentration of solid matter plays an important role in the alkali-activation process [156].

Fly ash contains high percentages of aluminium and amorphous silicon, making it suitable to produce geopolymers [157]. The steps of the chemical process producing geopolymers by alkaline activation of fly ash, according to Buchwald et al. [158], can be defined by the chemical reactions shown in Equations (12)–(17), specific to each constituent oxide of the individual fly ash, and the general chemical reaction according to Equation (18).

- hydration process for vitreous silica with a pH > 12:

SiO_2_ + 2OH^−^ → SiO^3−^ + H_2_O (12)

- hydration of Al_2_O_3_:

Al_2_O_3_ + 2OH^−^ → 2AlO^2−^ + H_2_O (13)

- reaction of CaO and MgO:

CaO + H_2_O → Ca^2+^ + 2OH^−^ (14)

- reaction of Na_2_O and K_2_O:

Na_2_O + H_2_O → 2Na^+^ + 2OH^−^ (15)

- the Fe_2_O_3_ reaction:

Fe_2_O_3_ + 3H_2_O → 2Fe^3+^ + 6OH^−^ (16)

- hydration of TiO_2_:

TiO_2_ + OH^−^ → HTiO^3−^ (17)

SiO_2_^.^αAl_2_O_3_^.^βCaO^.^γNa_2_O^.^δFe_2_O_3_^.^εTiO_2_ + (β + γ + 3δ)H_2_O + (2 + 2α + ε)OH → SiO_3_^2−^ + 2αAlO^2−^ + βCa^2+^ + 2γNa^+^ + 2δFe^3+^ + εHTiO^3−^ + (1 + α)H_2_O + 2(β + γ + 3δ) (18)

### 3.1. Physical-Mechanical Characteristics of Geopolymer Composites

The Si/Al ratio is a significant factor affecting the degree of crystallization and reaction with alkali activators [159], forming amorphous to semi-crystalline phases. Both polysialate-siloxo (Si/Al = 2) and polysialate-diloxo (Si/Al = 3) provided good strength of geopolymers. Polysialate-siloxo (Si/Al = 2) appears to be formed faster and has lower compressive strength than polysialate-diloxo (Si/Al = 3). The monomeric group of [SiO(OH)_3_]^−^, [SiO_2_(OH)_2_]^2−^ and [Al(OH)_4_]^−^ normally forms later than Si and Al species, because small aluminium silicate oligomers can enhance geopolymer formation [160]. Research tends to indicate that metakaolin-based geopolymers exhibit satisfactory compressive strength with a Si/Al ratio of 1.9 ÷ 3, while the appropriate ratio for fly-ash-based geopolymers is about 2 ÷ 4 [161,162]. Due to the large availability of fly ash, and the need to reintroduce it into the economic circuit, in line with the principles of the Circular Economy, much research has been directed towards making these geopolymers using fly ash. In this context, the issue of integrating affordable eco-efficient solutions into the value chains is a challenge, while combating climate change and reducing the negative impact on the environment by reducing waste and by-products from industry, reducing cement consumption and thus reducing greenhouse gas emissions, etc., while ensuring the development of advanced innovative materials. Class F fly ash has been identified as the most suitable raw material for geopolymer materials due to its reactivity and availability. The mass ratio of SiO_2_ and Al_2_O_3_ in Class F fly ash is between 1.7–4.0, while the amorphous content is generally higher than 50% [163,164,165]. Class C fly ash has a calcium content of 15–40%. It offers a different geopolymer structure compared to class F fly ash due to the increased calcium content. Temuujin et al. (2013) [165] reported that class C fly ash has self-cementing properties, which, through alkaline activation, allow it to harden at room temperature. Having a low calcium content, Class F fly ash, according to the literature studied, was preferred for producing geopolymer binders because the high amount of calcium may impact the polymerization process which may lead to changes in the microstructure of the final product [165,166,167,168,169].

During geopolymerization, fly ash reacts with the alkaline medium and specifically with aqueous solutions of polyisalates, leading to the formation of geopolymer materials comprising alumino-silicate-hydrate (A-S-H) gel [167]. Fly-ash-based geopolymers have demonstrated good mechanical strength and enhanced durability [167,168,170].

Several additions introduced at preparation in the geopolymer composite can have beneficial effects, improving the characteristics of the material in the fresh state but especially in the hardened state. Thus, literature indicates beneficial effects of nano-SiO_2_ in small amounts (max. 10 wt% relative to the amount of FA) on workability, polymerization reaction, mechanical strength and durability [171,172,173,174,175,176], and the use of MgO powder as an addition can improve aspects related to compressive strength, workability, drying shrinkage and porosity [177]. However, all these should be analyzed in relation to the oxide composition and specific characteristics of fly ash used as the main raw material.

The concentration of the alkali activator has a significant effect on the compressive strength of the geopolymer. The test age and the temperature at which curing (heat treatment) of the geopolymer takes place are other variables that influence the compressive strength of the geopolymer. However, with all the still existing uncertainties, research has shown that a sufficient concentration of activator is required during geopolymerization, as the NaOH concentration has a greater influence on the compressive strength values than on the curing time [178,179,180]. A common alkaline solution used for making fly-ash-based geopolymers consists of sodium silicate and sodium or potassium hydroxide, respectively, a solution with molar concentrations of sodium hydroxide between 7 and 10 M. The combination of NaOH and sodium silicate (Na_2_SiO_3_) is the most suitable for the alkaline activator because sodium silicate contains partially polymerized and dissolved silicon, which reacts more easily. Research shows that geopolymers with higher strength, for the same type of fly ash, are obtained when the ratio (Na_2_SiO_3_/NaOH) is between 0.67 and 1.00 [181], with a molar concentration of sodium hydroxide (NaOH) of at least 8–10 M [166,182].

In this direction, Al Bakri et al. [183] studied the effect of Na_2_SiO_3_/NaOH ratio on the compressive strength of fly-ash-based geopolymer, showing that by increasing the Na_2_SiO_3_/NaOH ratio from 0.6 to 1.00, the compressive strength increased, with the maximum compressive strength obtained for Na_2_SiO_3_/NaOH ratio equal to 1.00 [184,185]. Morsy et al. (2014) [186] studied the effect of Na_2_SiO_3_/NaOH ratios of 0.5, 1.0, 1.5, 2.0 and 2.5 on the strength of fly-ash-based geopolymer mortar. The highest value of compressive strength was obtained when the ratio was equal to 1.0 and increasing the Na_2_SiO_3_/NaOH ratio caused a decrease in compressive strength. Álvarez-Ayuso et al. (2008) [187] showed that when the NaOH concentration is higher, geopolymerization can be achieved even without soluble sodium silicate.

Rangan et al. [188] proposed 0.2 < Na_2_O/SiO_2_ < 0.28 and 15 < H_2_O/Na_2_O < 17.5 as optimum oxide ratios in the activation solution to achieve improved performance in geopolymer concrete. Provis et al. [189] stated that if the activator to binder mass ratio is between 0.6 ÷ 0.7 and the activator has a SiO_2_/Na_2_O ratio in the range of 1 ÷ 1.5, the resulting geopolymer binder imparts better mechanical properties. Another study showed that as the ratio of sodium hydroxide to sodium silicate increased from 1 to 2.5 and the molar concentration of NaOH increased from 8 to 16 M, the compressive strength of fly-ash-based geopolymers increased, i.e., the highest compressive strength was obtained for NaOH (16 M) concentration and sodium hydroxide/sodium silicate ratio in the range of 1.5 to 2.0 [190].

Vora and Dave [191] concluded that an increase in the sodium hydroxide/sodium silicate ratio and a higher molar concentration of sodium hydroxide result in higher compressive strength. However, Sindhunata et al. [192] reported that a soluble silicate content (SiO_2_/M_2_O > 2, where M is alkali ion) may reduce the reactivity in alkali-activated mixtures, as the concentration of cyclic silicate species may inhibit further condensation of aluminium ions. Therefore, higher concentration is judged to give rise to stronger ion pair formation and provide a more complete and faster polycondensation process of the particle interface [184,193], improving the dissolution of silicon and aluminium-containing materials in the presence of activators [194], but too high concentration could lead to an increase in the coagulated structure [195], causing lower workability with rapid curing behavior [196].

The mass ratio of alkaline solution to raw material is widely used in geopolymer synthesis to define both alkaline dosage and water content. In most cases, fly ash was used and the ratio was called alkali activator to fly ash ratio (AA/FA). Barbosa et al. [197] analyzed the effect of AA/FA ratio on strength development using 10 M NaOH solution as alkaline solution with AA/FA ratio = 0.34 ÷ 0.46. It was observed that the compressive strength increased when the AA/FA ratio increased to 0.40. Too high of an AA/FA ratio could lead to a decrease in concentration as more sodium carbonate was formed and obstructed the geopolymerization process [198]. It was found that, depending on the type of alumina-silicate source materials, the recommended AA/FA ratio could be between 0.35 ÷ 0.50 to have good compressive strength as well as good workability [199,200].

In terms of heat treatment temperature, research conducted on geopolymer paste and mortars for a temperature in the range (30 °C and 90 °C) demonstrated an increase in the overall geopolymerization reaction, leading to an increase in compressive strength from an early age [201]. However, exposure of the material to a treatment temperature above 90 °C will result in a geopolymer with a porous structure due to the rapid loss of water from the mixture, which will result in a possible decrease in mechanical performance [202]. Therefore, heat treatment is considered to improve mechanical properties, but the optimum temperature that improves the geopolymerization process and leads to the development of a suitable geopolymer microstructure is in the range 60 ÷ 75 °C [203].

It is very difficult to accurately identify all the factors influencing the properties of alkali-activated geopolymer materials and to quantify the influence of each. According to the literature, the main factors influencing the properties of geopolymer materials are: type of raw materials and their Si and Al percentage content; Si and Al ratio; type of alkaline activator used; molarity of NaOH or KOH solution used; Na_2_SiO_3_/NaOH mass ratio; duration and temperature of heat treatment; test age of the geopolymer material [204,205,206,207,208,209,210,211,212,213,214].

In the case of geopolymers, temperature and curing time play a significant role in the kinetics of the unfolding of chemical reactions: at low temperatures the evaporation of geopolymer precursors and water molecules occur simultaneously, preventing the formation of voids and cracks within the material, thus increasing the compressive strength [215,216]. This suggests that a longer curing time at low temperatures is preferable for the synthesis of geopolymer with higher compressive strength.

### 3.2. Influence of TiO_2_ Adding Nanoparticles in Geopolymer Composites

It should be noted that, as reported by several research [217,218,219,220,221,222], a problem in producing these composites is the way of incorporation of NT, as this is relatively difficult to ensure a homogeneous dispersion, NT tending to agglomerate. Another controversy is in terms of self-cleaning capacity, with Guerrero et al. [220] indicating that 1 wt% NT is sufficient and Yang et al. [221] indicating an optimum requirement of 10 wt% NT.

Geopolymer concrete has been used in various applications such as waste management [205], civil engineering [222,223], cements and concretes [224,225], building retrofit [226] and as an alternative binder to replace OPC [226,227,228]. Applications have been extended to pavements [229,230,231,232] and building facades [223,229,232] and special function coatings [233]. Aguirre-Guerrero et al. [234] explored the potential of hybrid geopolymer coatings as a protective coating for reinforced concrete structures subjected to a marine environment. Sikora et al. [217] studied the applicability of geopolymer mortar as a coating to protect concrete from chemical attack and corrosion. Self-cleaning facades are gaining momentum in the construction industry. The applicability of self-cleaning facades is due to improved performance in brightness and reflectivity without the need for frequent maintenance [218,219].

Research reports on the influence of NT introduction in the geopolymer matrix on micro- and macro-structural characteristics, physical-mechanical and durability performances are found in a limited number of literatures. The literature indicates possibilities to induce self-healing capacity on geopolymeric materials using ZnO, TiO_2_, WO_3_ or Fe_2_O_3_ nanoparticles, with preference for NT and ZnO due to their high stability and low toxicity [9,235,236,237,238,239,240,241,242]. However, although they represent an environmentally friendly alternative in the construction industry, the properties of geopolymer materials with self-healing capacity are not yet sufficiently investigated so that they can be exploited.

#### 3.2.1. NT Influence on Geopolymer Paste Properties

Experimental research conducted by Ambikakumari Sanalkumar and Yang [243] for geopolymer produced using metakaolin and containing 1–10% nano-TiO_2_ (mass percentages reported to the mass of metakaolin) showed that the addition of nano-TiO_2_ induces reduction in the flowability of the geopolymer paste. Such behavior could be associated with the high surface area of nano-TiO_2_ particles, which creates the water demand of the mixtures [244]. In addition, the existence of very fine particles brings stronger cohesive van der Waals forces inside the particles, resulting in flocculated and agglomerated particles, which also makes the fluidity of the geopolymer paste in its fresh state become lower. Specifically, the fluidity of the fresh geopolymer paste decreases, relative to the fluidity of the control fresh geopolymer paste (without nanoTiO_2_) by 3.22%; 5.65%; 25%, and by 37% when 1%, 2%, 5% and 10% nanoTiO_2_ are introduced into the geopolymer paste (mass percentages relative to the amount of metakaolin).

Research by Duan et al. showed that in the case of replacing 1%, 3% or 5% fly ash with nano-TiO_2_ (massive percentages relative to the amount of ash), the effects are also significant. Thus, a reduction in the workability of the nano-TiO_2_-containing geopolymer is reported, the workability decreasing by 7.9% for the case of 1% nanoTiO, and by up to 20% if 5% fly ash is substituted with nano-TiO_2_.

#### 3.2.2. Influence of NT on the Properties of Geopolymer in Hardened State

Mechanical properties: Several studies have reported that the inclusion of TiO_2_ in geopolymer binders induces certain effects on the mechanical properties of the resulting binder. Duan et al. [245] conducted a study to examine the impact of nano-TiO_2_ inclusion on the physical and mechanical properties of a fly-ash-based geopolymer. The study showed that the incorporation of nano-TiO_2_ particles increases compressive strength and carbonation resistance, reduces the drying shrinkage of the geopolymer [245]. The addition of nano-TiO_2_ densifies the microstructure of the geopolymer matrix, in contradiction to a study [244] that reported that the inclusion of micro TiO_2_ particles does not improve the mechanical properties of the geopolymer.

In terms of density, experimental research [244] reported an increase of up to 12% in density and a reduction of up to 41% in the volume of pore sizes ranging from 2 nm to 5 μm, concomitant with an increase in nano-TiO_2_ content. These manifestations are thought to be due to the filling of fine pores by TiO_2_ nanoparticles, thus inducing changes in the geopolymer at the microstructural level. Because of the densification of the material, but not only, as expected, the compressive strength at 7 days was improved by up to 41% (compared to the compressive strength of the control sample without nano-TiO_2_) as the amount of nano-TiO_2_ used was increased. Samples stored and tested at longer than 7 days (14 days, 28 days) also showed increases in compressive strength but at lower levels. This behavior shows that, unlike cementitious composites, where the increase in compressive strength occurs as a result of hydration-hydrolysis processes taking place over time (28 days), in the case of geopolymer binders the strength performance is obtained as a result of aluminosilicate dissolution processes and formation of the specific three-dimensional structure, and a densification of the material induces beneficial effects.

Research by Duan et al. showed that in the case of replacing 1%, 3% or 5% fly ash with nano-TiO_2_ (massive percentages relative to the amount of ash), in terms of drying shrinkage, the presence of nano-TiO_2_ in the geopolymer paste has a beneficial effect in reducing this indicator. This behavior is attributed to the possibility of filling the pores of the geopolymer paste with nanoparticles, which leads to densification of the material.

In terms of the performance of the hardened composite, there is an increase in the compressive strength at 7 days after peening, compared to the control sample, by more than 4% in the case of substitution of 1% fly ash with nanoTiO_2_ and by more than 17% in the case of 5% nano-TiO_2_. This increase in compressive strength is also evident when testing specimens at lower or higher ages, i.e., 1 day, 3 days after pouring or 28, 56, even 90 days after pouring. However, in agreement with other reports in the literature, the intensity of the effect on the compressive strength at early ages is noted, with the highest increases in compressive strength recorded compared to the control sample 24 h after casting, respectively, 7.1% for 1% nano-TiO_2_ and 51% for 5% nano-TiO_2_, a sign that these particles act as a densification spinner and induce microstructural changes.

According to Zulkifli et al. [246], the geopolymer made from metakaolin with NT content shows a much more homogeneous, compact microstructure with low porosity compared to the control sample made without NT. Syamsidar et al. [247] present results on a geopolymer material made by heat treatment at 50 °C based on class C fly ash from Bosowa Power Plant Jeneponto, Suth Sulawesi alkali activated, SiO_2_/Al_2_O_3_ = 3; Na_2_O/SiO_2_ = 2 and H_2_O/Na_2_O = 10, in which 5%, 10% or 15% NT (mass percentage relative to the amount of fly ash) were introduced. Tests on specimens matured up to 7 days showed a much more compact and smooth surface appearance, without apparent porosity or surface defects, with apparent density increasing slightly with increasing %NT (2.85%, 3.1% and 4.76% increase in apparent density for 5%, 10% and 15% NT samples, compared to the apparent density of the control—0% NT). The compressive strength did not show a continuous increase as %NT increased, with a maximum recorded for the 10% NT composite, i.e., an increase of 12.36% for the 5%NT sample, 53.74% for the 10%NT sample and 45.64% for the 15%NT sample, suggesting the need to identify the optimal NT content interval in the geopolymer matrix in order to obtain the best compressive strength. XRD diffractograms performed for the NT geopolymer showed that, at microstructural level and compared to the control geopolymer (0%NT), no new crystallization phases are identified in the structure, therefore NT added during the preparation does not react with the constituents of the geopolymer, like the behavior of NT in cementitious matrices. Supporting the results obtained in the compressive strength evaluation, SEM analysis of the specimens indicates numerical reduction and decrease in microcracks opening for the case of NT specimens compared to the control geopolymer. Additionally, SEM analysis indicates a good adherence of the NT in the geopolymer matrix. In terms of resistance to H_2_SO_4_ action (1M, immersion 3 days), the strong reaction with CaO in fly ash is indicated with the formation of gypsum crystals (CaSO_4_-2H_2_O), which is favored by the existence of NT in the geopolymer matrix. The formation of these crystals will induce internal stresses in the composite matrix which will favor the degradation process of the material, therefore, the use of a CaO-rich fly ash is not favorable for the use of a geopolymer composite with NT if it is intended for use in an acidic environment. Regarding the self-healing capacity, it was analyzed on specimens immersed in red clay solution showing that after removal from the solution, the surface of the specimens remained clean, without adherent red clay particles.

Guzman-Aponte et al. [248] showed that the inclusion of up to 10 wt.% NT did not influence the development of calcium silicate hydrate gel but did not indicate in detail the effect of NT addition on the physico-mechanical properties of GP.

Duan et al. [245], analyzing the influence of NT on the physico-mechanical characteristics of geopolymer paste prepared by alkaline activation of fly ash (alkaline activator prepared based on Na_2_SiO_3_ and NaOH), showed that 5 wt%, NT relative to the amount of fly ash, compared to the geopolymer control sample without NT, contributes to an increase in the compressive strength both at early ages and 28 days after casting, to obtain a more compact microstructure with less microcracks and improves the carbonation resistance of the composite. Duan et al. contribute by their research, and given that, reports on the carbonation resistance of nano-TiO_2_-containing geopolymer are rare. Thus, it is shown that, as a result of these microstructurally induced changes, the depth of carbonation decreases as the number of nanoparticles substituting fly ash increases, the effect being even more evident compared to the control geopolymer, the longer the duration of exposure to carbonation. These results agree with the results reported by Sastry et al. [249], which indicate the increase in compressive strength of alkaline-activated fly-ash-based geopolymer for 2.5 wt% NT and Yang et al. respectively, [248] who, for the case of geopolymer made by alkaline activation of slag indicate the role of NT on several factors, namely, on geopolymer formation reactions, reduction in microcracks, cracks and improvement of compaction at the microstructural level.

Subaer et al. [250] analyze the thermo-mechanical properties of geopolymer composites made from alkali-activated metakaolin with soil. Na_2_SiO_3_ + NaOH, dispersive reinforced with 1–2 wt% carbon fibres and with NT applied as surface coating. The results showed that the geopolymer represents a good adhesion incorporating NT, but since NT are not included in the composite matrix and represent only a surface coating, their influence on the thermo-mechanical performance of the composite is not noticeable, carbon fibers having a more significant influence.

Mohamed et al., analysing the effect of TiO_2_ on the performance of alkaline activated meta-halosite based geopolymers with potassium hydroxide and potassium silicate-based activator, indicate that additions of 2.5%, 5%, 7.5% or 10% nanoparticles (mass percentage) reduce the total pore size (total porosity) by up to 49% compared to the control geopolymer, proportional to the amount of NT used. An increase in tensile strength of up to 78% is also reported, proportional to the amount of nano-TiO_2_ used. All this leads, in agreement with other reports in the literature, to the conclusion that NT has a beneficial densification effect at the microstructural level, but it is noted that rutile TiO has a stronger effect than anatase TiO.

SEM-EDS analyses reported by Bonilla et al. [251] indicate the possibility of obtaining more homogeneous, smooth, compact surfaces with a reduced number of cracks compared to the control sample. In terms of physical-mechanical properties, a slight increase in density was observed, but, probably due to the heterogeneous distribution and agglomeration of nanoparticles both in the NT and nano-ZnO cases, the compressive strength decreased significantly by more than 2.5 times compared to the control.

Self-cleaning capacity and biocidal capacity: The self-cleaning performance of nano-TiO_2_ modified geopolymer as a potential building material has been rarely reported in the literature [248]. However, as with cementitious composites, this self-cleaning ability is the sum of two main mechanisms: the ability to modify the surface’s hydrophilicity and the ability to decompose organic molecules and even microorganism cells through redox reactions. The most common methods of evaluation from this point of view are oriented towards the evaluation of surface hydrophilicity (induction of superhydrophilicity of the surface), the evaluation of the decolorization capacity of rhodamine B or methylene blue, respectively, the evaluation of resistance in the presence of an environment contaminated with microorganisms. Experimental research by Ambikakumari Sanalkumar and Yang [243] showed an improvement of up to 15% in total solar reflectance (TSR) for nanoTiO_2_-containing geopolymers compared to the control sample. Additionally, an improvement in hydrophilicity and surface self-healing ability is indicated and obtained on the one hand, as a result of the photoactivation of nano-TiO_2_ and, on the other hand, as a result of the use of NaOH in the alkaline activation of the raw materials to obtain the geopolymer, since it is known that NaOH has a strong decomposition effect on organic molecules [252,253].

In agreement with Loh et al. [254], it is estimated that incorporation of NT into fly ash or kaolin-based composites has the effect of increasing the photocatalytic activity of NT. Moreover, even in the absence of light, based on the MB decolorization test and tests using microbiological techniques, antifungal properties were demonstrated.

Zailan et al. [255] report a review on the induction of self-cleaning ability by introducing 2.5%, 5% and 7.5% NT into the geopolymer matrix and evaluating/demonstrating this performance using *rhodamine B* (RhB) and *methylene blue* (MB) staining tests, respectively. They also analyze the influence of ZnO nanoparticles on the performance of geopolymer prepared based on fly ash (FA) class F from CIMA plant Perlis, Malaysia and alkaline activator based on Na_2_SiO:NaOH, 12 M = 2.5:1.0 in which 2.5; 5; 7.5 and 10, wt%, ZnO nanoparticles were introduced. The results of the research on specimens matured up to 28 days showed a reduction of the compressive strength by approx. 29–54% compared to the control sample (0% ZnO nanoparticles), depending on the amount of nano-ZnO used, a behavior also supported by microstructural analysis by XRD and Sem which reveals changes in the crystallization phases. In terms of surface self-healing ability, based on the methylene blue (MB) staining test, a continuously increasing stain discoloration is recorded over time (over the evaluation time of 150 min UV exposure) and in relation to the amount of nano-ZnO used. This phenomenon is explained by a mechanism similar to the one presented for NT and confirms the possibility of inducing photocatalytic character on geopolymer not only using NT but also using nano-ZnO.

In consensus, Min et al. [256], Gasca-Tirado et al. [257], Zhang and Liu [258] and Luhar et al. [259], Kaya et al. [260], Chen et al. [261] indicate good results in terms of self-healing ability by photocatalytically activated degradation of methylene blue (MB) stains—Zhang and Liu indicating a 93% reduction of methylene blue stain staining for the case of photoactivated alkaline activated fly-ash-based NT-containing geopolymer surface within the first 6 h. However, research shows that part of the reduction in the degree of staining is due to the absorption of the dye into the geopolymer mass, and the rest is due to the degradation of the dye under the action of photoactivated NT [258,259]. Additionally, some research even indicates that GP itself has its own antimicrobial, antifungal and decolorizing capacity of staining substances, but the addition of NT has a strong increasing effect on these properties [254,260,262,263].

Yang et al. [264] report the effects of introducing 10 wt% NT on the performance of a geopolymer matrix made from fly ash from Shenhua Junggar Energy Corporation in Junggar, Inner Mongolia, China. Experimental XRD, SEM, BET analysis and photocatalytic activity results indicated the possibility of uniform distribution of NT in the geopolymer matrix and the influence of NT distribution mode on the specific geopolymer–NT surface area and photoactivity, i.e., the decolorization capacity of MB, which collapsed with increasing distribution homogeneity.

Alouani et al. [265] investigated the ability of geopolymer material produced by alkaline activation of metakaolin as an adsorbent to remove methylene blue. Strini et al. [266] showed that for 3 wt% NT added in geopolymer paste made from fly ash and metakaolin, geopolymer binders can be effective matrices to support photocatalytic activation of NT and induce specific material properties. Relating also to self-cleaning properties, Wang et al. [267] show that 5 wt% NT would be the optimum percentage for maximum MB decolorization effect, but research is insufficient because GP performance is influenced by several factors, as shown in the head above. Finally, and in terms of the influence on the physical-mechanical properties of GP, it is still controversial how much NT is introduced into the GP paste to achieve optimal performance.

Qin et al. [268] indicate the possibility of making superhydrophobic geopolymer surfaces by alkaline activation of blast furnace slag (also Si and Al oxide supplier waste) with reported good results in terms of hydrophobicity and therefore durability, including the fouling resistance capability of the material. In a similar direction of research development, Chindaprasirt et al. [269] indicate the possibility of inducing superhydrophobicity and self-cleaning capability of the surface of a geopolymer made based on alkali-activated fly ash, Na_2_SiO_3_/NaOH = 2, but in this case which benefited from a polymeric surface coating. Permatasari et al. [270] indicate the possibility of inducing self-healing performance for the surface of a geopolymer made from Gowa Regency soil deposit laterite, to which a thin film of NT solution coating was sprayed. This method allows for inducing a self-cleaning character, without influencing the flexural strength, with the mention that, once this film is destroyed, the self-cleaning capacity is lost.

In terms of bactericidal effect on *K. pneumoniae* and *P. aeruginosa*, Bonilla et al. [271] analyze a geopolymer composite based on powder material consisting of alumino-silicate precursors (85%) and Portland cement (15%), alkaline activated and containing 5 wt%, 10 wt% NT, 5 wt%, 10 wt% nano-ZnO, respectively. Research results demonstrate the development of inhibition halos and bactericidal effect such as a gentamicin antibiotic for both composite paste and composite mortar. In the same paper, the authors also indicate the self-cleaning effect developed by photocativation, an effect that causes rhodamine B, RhB, to decolorize 76.4% after 24 h for NT and over 98% for nano-ZnO.

## 4. Future Perspectives

At present, possibilities for increasing the specific effect induced by NT in both cementitious and alkali-activated geopolymer composites are reported in the literature. Thus, due to the specificity of NT being a wide bandgap semiconductor, i.e., 3.2 eV vs. the normal hydrogen electrode (NHE), it is indicated that through photoactivation, electrons jump from the valence band to the conduction band but a rapid charge recombination, i.e., a return of electrons to the valence band, occurs. This behavior is not beneficial from the point of view of the electron lifetime in the conduction band and, therefore, the performance of the composite by photoactivation. In order to improve these performances of the composite, acquired by photoactivation, in terms of increasing the charge separation efficiency (production of electrons and holes) and implicitly, the duration of manifestation of the self-cleaning effect as well as the biocidal effect, after the light source is removed, the literature indicates the possibility of introducing carbon-based materials (graphene, graphene oxide, carbon particles, fullerenes, which function as absorbers, acceptors and electron carriers) [271,272,273,274,275,276,277,278,279,280,281]. Therefore, the composites will show an increased absorption of light also in the visible spectrum, this technique becoming a possibility to improve the performance of the composites, i.e., to induce the possibility of photoactivation also with incident rays with wavelength in the visible spectrum. Hamidi and Aslani [22,282,283,284,285,286,287] indicated that the efficiency of the NT photoactivation process is influenced by a number of factors, the most important of which are: efficient absorption of sunlight, separation of products from the photocatalyst surface, rapid charge separation after light absorption to prevent electron-hole recombination, compatibility of the redox potentials of the valence band hole and conduction band electron with those of the donor and acceptor species, and long-term stability of the photocatalyst. Furthermore, research has shown [22,287,288,289] that the photocatalytic properties of TiO_2_ are influenced by particle size, surface area, pore volume, surface hydroxyl content and degree of crystallinity. Crystallinity is an important factor contributing to the high photoactivity, as the presence of an amorphous phase would facilitate the recombination of photoexcited electrons and holes. Therefore, on a case-by-case basis, the optimal dosage of NT in cementitious composites or geopolymer materials is strongly influenced by a combination of all these factors.

This paper addresses a current research topic, with the aim of contributing to the creation of a favorable framework for the development and validation of materials with specific performances of the “smart-innovative” concept (the ability of a composite intended for the construction sector to harden in the absence of cement, while at the same exploiting the self-cleaning capacity), thus opening up new opportunities for the immediate exploitation and valorization of waste and by-products in accordance with current international environmental and sustainable development policies.

The multi- and transdisciplinary as well as eco-innovative character derives from the strongly applicative-exploratory approach of a research field involving on the one hand aspects of sustainable development of the built environment, and on the other hand specific aspects responding to the European requirements of environmental impact and population health. At the same time, a cross-cutting connection is made between the industries generating waste/industrial by-products (energy, metallurgy, natural resource processing) and the construction industry, creating a favorable framework for the implementation of emerging technologies with a high degree of novelty and the possibility of obtaining innovative intelligent products with the potential to advance in global value-added chains. Moreover, the exploitation of the specific performance of nanomaterials provides a link with other areas (safety, hygiene, health) by contributing to the production of knowledge needed to develop technologies that induce a high degree of safety in terms of surface hygiene, with high resistance to the development of biological films of microorganisms (moulds, lichens, algae, bacteria). Consequently, a synergistic connection is achieved between applied research activities aimed at (1) developing innovative composite materials for the sustainable development of the built environment; (2) developing materials with low environmental impact; (3) expanding the range of possibilities for introducing industrial wastes and by-products into the economic circuit; (4) developing innovative intelligent materials with self-cleaning capacity and increased resistance to biological agents; and (5) developing effective solutions for increasing safety in terms of public health.

Therefore, the contribution of this review responds to the need to connect current research to the European and global scientific frontier, in line with societal challenges related to responsible consumption and production; combating climate change and reducing negative environmental impacts.

## 5. Conclusions

The aim of this study was to summarize, as far as possible in parallel, the current state of research into the development of high-performance, self-healing composites with low environmental impact, or which allow the reduction in environmental impact by using industrial waste or by-products as raw materials. Thus, an analysis focused on the field of cementitious composites based on Portland cement vs. geopolymer composites based on alkali-activated fly ash, with NT content in mass, without analysing other materials, (non-Portland cements, lime mortars, lime and cement mortars, hydraulic lime mortars modified with TiO_2_).

Based on the above presented, in general, the following can be said:

- in the current context, where the need to identify sustainable development solutions is imperative, the development of innovative materials contributes to the creation of a favorable framework for increasing the implementation of the principles of the Circular Economy, reducing environmental impact and increasing sustainability in the construction sector;
- innovative directions in this respect, still niche, are the development of cementitious composites or geopolymer composites that include nanoparticles in the matrix, the most used being NT;
- in parallel, the development of geopolymer materials allows for the reuse of waste or industrial by-products which contributes to reducing the environmental impact of other industries.
- In terms of cementitious composites, studies conducted to date have shown that:
- inducing exceptional properties by exploiting specific features of the nanoparticles embedded in the composite matrix has already proven to be a possible way forward;
- to date, although the research carried out is encouraging, there are several controversies and uncertainties, which point to further research;
- the introduction of NT into the cementitious matrix has consequences on the physical-mechanical performance, durability or resistance performance to the action of micro-organisms, improving them;
- the results of the research carried out at microstructural level, corroborated and reflected by the results of the research carried out at macrostructural level, indicate the need for in-depth analysis so as to gain a thorough knowledge of the mechanisms underlying the phenomenon and to be able to determine more easily and more precisely the optimal quantity of nanoparticles and the way in which they are introduced during preparation, so as to achieve good performance in terms of physical-mechanical properties, self-healing capacity and increased surface hygiene.
- In terms of geopolymer materials, studies conducted so far have shown that:
- the field of self-healing geopolymers is an area of interest, but one that has been addressed only in recent years;
- so far, a number of results are reported, but there is still some controversy about the mechanisms of the geopolymerization reaction, the influence of a significant number of factors (e.g., type and oxidative composition of the raw material, characteristics of the alkaline activator, existence or not of nanoparticles or the type of nanoparticles used, working temperature, etc.) on the physical-mechanical performances, which have been studied more intensively, but also on some performances of durability, self-healing capacity, resistance to the action of microorganisms, etc.

The scientific contribution of the paper derives from the cumulative approach, in parallel, presenting possibilities of integrating smart functions within the eco-friendly function for the development of technologies and materials for the construction sector, in the context of applying and integrating the principles of the Circular Economy as a tool for Sustainable Development. Therefore, the general objective of the work has been set in order to respond to the need to connect current research to the European and global scientific frontier, in line with societal challenges related to responsible consumption and production through the efficient use of resources and raw materials, having as basic benchmarks the elements of scientific novelty in the field of smart-eco-innovative composite materials.

## Data Availability

Not applicable.
